# Primary leiomyosarcoma of the submandibular gland: a case report

**DOI:** 10.1186/s12907-015-0022-4

**Published:** 2015-12-15

**Authors:** Mohamed Reda El Ochi, Hafsa Chahdi, Issam Rharrassi, Abderrahman Albouzidi, Mohamed Oukabli

**Affiliations:** Department of Pathology, Mohamed V Military Hospital, Hay Riad, Rabat Morocco

**Keywords:** Leiomyosarcoma, Salivary, Submandibulary, Gland

## Abstract

**Background:**

Leiomyosarcoma is a rare malignant mesenchymal tumor that represents 5–7 % of all soft tissue sarcomas. The occurrence of this tumor in the salivary glands is exceptional. Only five cases are reported in the submandibular gland.

**Case presentation:**

A 65-year-old white Moroccan woman presented with a history of progressive right submandibular swelling which had grown over a period of 8 months. Clinical examination showed a submandibular painless, mobile and hard mass without cervical lymphadenopathy. Ultrasonography and computed tomography revealed a solid and heterogeneous mass measuring 4 × 2 cm involving the submandibular gland. A resection of the gland was performed. Pathological findings were consistent with primary leiomyosarcoma of the submandibular gland. No recurrence occurred after two months of follow-up.

**Conclusion:**

Primary leiomyosarcoma of the submandibular gland is an extremely rare mesenchymal tumor. Clinical and radiological features are not specific. Differential diagnosis includes metastatic leiomyosarcoma and gastrointestinal stromal tumor, myoepithelioma, sarcomatoid carcinoma, melanoma and other sarcomas. Little is known about the overall biologic behavior of this tumor, but the prognosis seems to be poor.

## Background

Primary sarcomas of the salivary glands are rare accounting for 0,3–1,5 % of salivary gland neoplasms and 1,5–2,3 % of malignant salivary tumors [1]. They arise mainly in the parotid gland and only in 10 % of cases in the submandibular gland [2].

Leiomyosarcoma is a malignant mesenchymal tumor with smooth muscles origin that represents 5–7 % of all soft tissue sarcomas [3]. The occurrence of this tumor in the salivary glands is exceptional [2, 4].

Five cases of primary leiomyosarcoma of the submandibular gland are reported. We describe here an additional case.

## Case presentation

### Clinical history

A 65 year-old white woman, without clinical antecedents, presented with a history of progressive right submandibular swelling which had grown over a period of 8 months. Clinical examination showed a submandibular painless, mobile and hard mass measuring 4 × 2 cm without cervical lymphadenopathy.

### Radiologic and histopathologic findings

Ultrasonography and computed tomography revealed a solid and heterogeneous mass measuring 4 x 2 cm involving the right submandibular gland. There was no cervical lymphadenopathy. A resection of the right submandibular gland was performed.

**Fig. 1:**
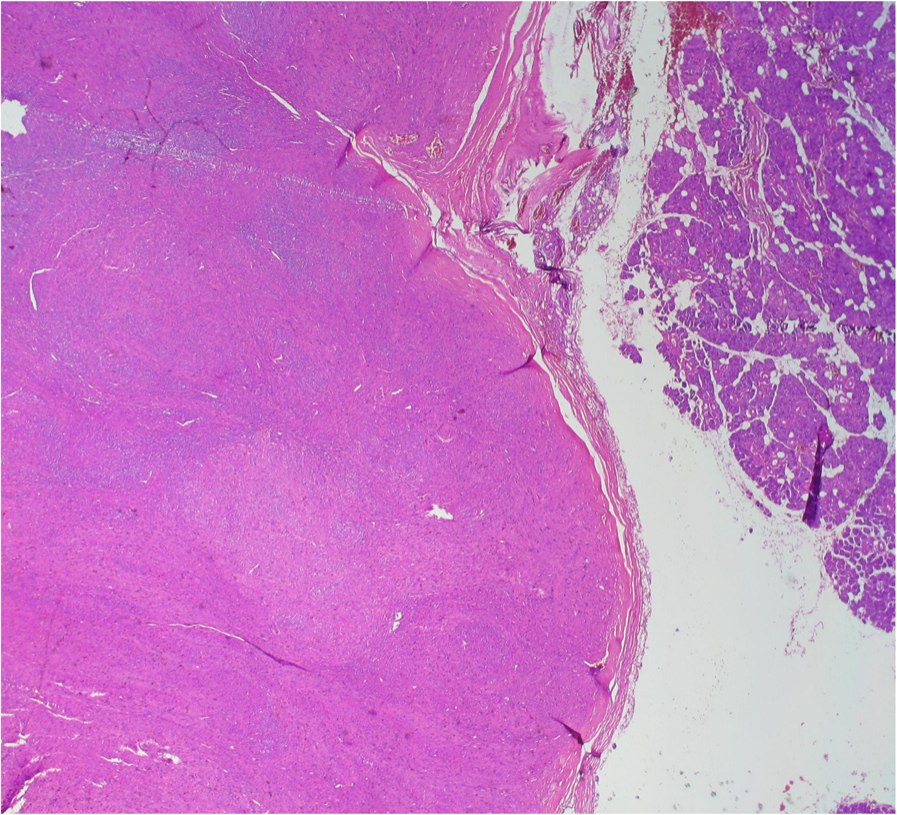
Salivary parenchyma harboring a well-circumscribed, fascicular proliferation of spindle shaped cells (hematoxylin and eosin stain, original magnification × 25)

**Fig. 2:**
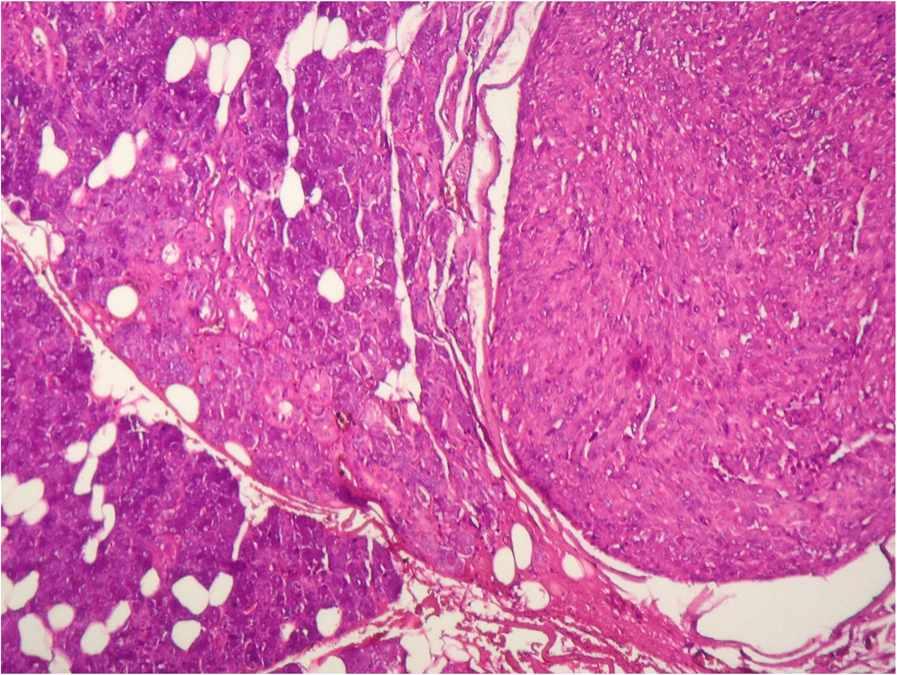
Tumor cells infiltrating the adjacent parenchyma (hematoxylin and eosin stain, original magnification × 200)

**Fig. 3:**
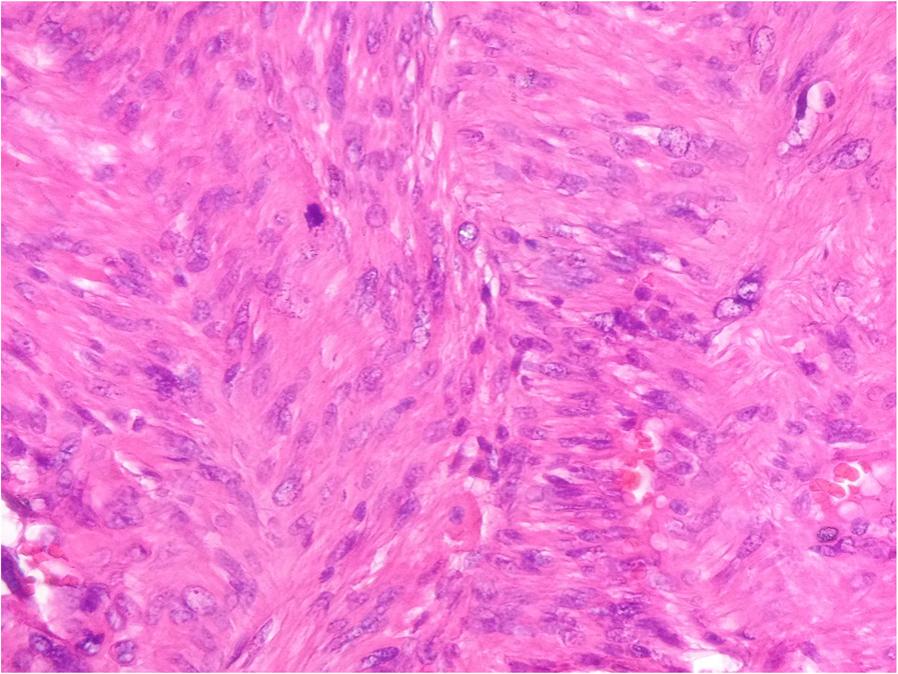
Tumor cells showing mild nuclear atypia with multinucleated giant cells and mitosis (hematoxylin and eosin stain, original magnification × 400)

**Fig. 4:**
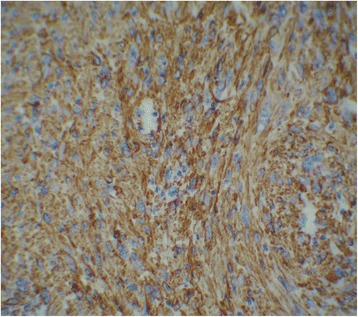
H-caldesmon positivity of the tumor cells (original magnification × 400)

Macroscopically, the specimen measured 6 x 4 x 2 cm. The tumor appeared as a grey white, hard mass and 4 × 2 cm in dimension. Histological examination showed intersecting fascicles of spindle cells (Fig. 1) with ample amount of eosinophilic cytoplasm and elongated nuclei with dispersed chromatin. The cells presented high mitotic activity (5 per 10 high-power fields) and foci of severe atypia without necrosis (Fig. 2). The adjacent salivary parenchyma was infiltrated by tumoral cells (Fig. 3). No areas of epithelial component were identified despite extensive sampling. Immunoreactivity with anti smooth muscle actin and H-caldesmon (Fig. 4) antibodies was found. Desmin, S-100 protein, CD34, CD31, CD117, and pancytokeratin were all negative. Thus, a diagnosis of leiomyosarcoma of the submandibular gland grade I FNCLCC (French Fédération Nationale des Centres de Lutte Contre le Cancer) was established. The post-operative course was uneventful. The thoracoabdominal computed tomography, performed subsequently, showed no distant tumor. The patient is dowing well without any evidence of recurrences or metastases after two months of follow-up.

## Discussion

Primary sarcomas of the salivary glands are rare accounting for 0,3–1,5 % of salivary gland neoplasms and 1,5–2,3 % of malignant salivary tumors [1]. Primary leiomyosarcomas of the head and neck are uncommon representing 3–10 % of leiomyosarcomas [5] and usually occur between 40–50 years old [6]. They arise mainly in the parotid gland and only 5 cases are reported in the submandibular gland in patients aged above 60 years [1, 2, 7, 8]. Tumors with the following criteria are considered primary salivary gland sarcoma: 1) The patient must not have, or have had, a sarcoma elsewhere; 2) a metastasis to the gland from malignancies of the skin or mucosa of the upper aerodigestive tract must be excluded; 3) the gross and microscopic appearances should be consistent with a primary origin, rather than invasion from the adjacent soft tissues; and 4) within the limits of the microscopic study of multiple sections, carcinosarcoma has to be excluded [9].

Clinical features are not specific with a painless and progressive mass being the most significant finding [10–12]. Radiological investigations allow evaluation of the extent of the tumor and assessment of the regional lymph nodes [5, 13].

Microscopically, leiomyosarcoma shows a proliferation of intersecting fascicles of spindle cells with ample amount of eosinophilic cytoplasm and elongated nuclei with dispersed chromatin [7]. Mitotic rate is superior to 2 mitosis per 10 high-power fields [5, 14]. Foci of pleomorphism and/or necrosis can be found [7]. Immunohistochemistry demonstrates positive staining of the tumor cells for smooth muscle actin and H-caldesmon [15]. Desmin can be either positive or negative [5, 14]. S-100 protein, CD34, CD31, CD117, and pancytokeratin are generally negative [15]; these findings play an important role in excluding myoepithelioma, sarcomatoid carcinoma, melanoma, metastatic gastrointestinal stromal tumor, and other sarcomas [5, 7, 16]. However, the main differential diagnosis is metastatic leiomyosarcoma from other malignancies of head and neck sites, soft tissues and uterine corpus; thus, clinical informations and imaging studies are very important [7].

Staging of leiomyosarcoma of the head and neck includes chest and abdominal computed tomography and bone scintigraphy [7].

The management is mainly surgical. Complete surgical excision with clear margins is the mainstay of the treatment [7, 12] being associated with low local recurrence and longer survival [7]. Lymph node metastases are exceptional in sarcomas so that lymphadenectomy is not indicated [7]. Adjuvant radiotherapy is recommended for high grade sarcoma, large tumour, and close or positive surgical margins [2, 10, 12, 13]. Chemotherapy is indicated for patients with inoperable tumors, local recurrence or distant metastases [2].

The prognosis seems to related to the size (greater or less than 3 cm), grade and site of the tumor and the quality of surgery [7, 9]. For major salivary gland, the prognosis is poor [2, 8].

In leiomyosarcoma of the head and neck, the five-year survival is 23 % [2]. Recurrences are frequent and occur in 40–60 % of cases [5]. Metastases are rare and occur mainly in lung, bone and central nervous system [12].

## Conclusion

In summary, primary leiomysarcoma of the mandibular gland is a rare and aggressive mesenchymal tumor that makes several problems in the differential diagnosis. An accurate diagnosis is imperative because of its poor prognosis. In our case, clinical informations and imaging studies revealed no other tumors.

## Consent

Written informed consent was obtained from the patient for publication of this Case Report and any accompanying images. A copy of the written consent is available for review by the Editor-in-Chief of this journal.
